# Effects of Shenxiang Suhe Pill on coronary heart disease complicated with nonalcoholic fatty liver disease: A case–control study

**DOI:** 10.1097/MD.0000000000031525

**Published:** 2022-12-09

**Authors:** Jie Ni, Chen Chen, Jiake Tang, Siqi Hu, Yao You, Shenghui Zhang, Jingjie Jiang, Chunyi Wang, Wen Wen, Xingwei Zhang, Mingwei Wang

**Affiliations:** a Hangzhou Institute of Cardiovascular Diseases, Affiliated Hospital of Hangzhou Normal University, Hangzhou, China.

**Keywords:** coronary heart disease, nonalcoholic fatty liver disease, Shenxiang Suhe Pill

## Abstract

To observe the clinical effect of Shenxiang Suhe Pill in the treatment of coronary heart disease (CHD) patients with nonalcoholic fatty liver disease (NAFLD).

**Methods:**

56 CHD patients with NAFLD were randomly divided into an experimental group and control group. The control group was treated by conventional western medicines, while the experimental group was given Shenxiang Suhe Pill in addition to the treatment of the control group. Both groups were treated for 12 weeks. Before treatment and after 12 weeks of treatment, the clinical efficacy indices of the 2 groups were evaluated, including transient elastic B-ultrasound (Fibroscan), controlled attenuation parameter (CAP), alanine aminotransferase (ALT), aspartate aminotransferase (AST), low density lipoprotein cholesterol, high density lipoprotein cholesterol, triglyceride (TG), total cholesterol, high sensitivity-reactive protein (hs-CRP) and lactate dehydrogenase (LDH).

**Results:**

Compared with the control group, the CAP value of the experimental group decreased more significantly, and the severity classification of NAFLD was also significantly improved (*P* < .05). LDH and hs-CRP in the experimental group decreased after treatment (*P* < .05). TG and high density lipoprotein cholesterol indicators improved more in the experimental group than in the control group (*P* < .05). ALT and AST in neither group showed significant change (*P* > .05).

**Conclusion:**

Shenxiang Suhe Pills has a significant overall curative effect in the treatment of patients with CHD complicated with NAFLD. It can reduce liver lipid deposition, reduce the severity of NAFLD, and has lipid-lowering and anti -inflammatory effects.

## 1. Introduction

Being a common cardiovascular disease (CVD), coronary heart disease (CHD) seriously threatens human life and health.^[1]^ Currently, there are approximately 290 million patients with CVDs and 11 million CHD patients in China, the numbers have increased significantly compared with previous years. Therefore, preventing and treating CHD has become ever more practical.^[2]^ Nonalcoholic fatty liver disease (NAFLD) is a syndrome characterized by fatty deposits. In addition to causing liver disability and death, NAFLD is also closely associated with high incidence of CVD, metabolic syndrome (MetS), and type 2 diabetes.^[3]^

To date, there is no efficacious drug for treating NAFLD and the mainstream treatments include lifestyle intervention, antioxidants, anti-inflammatory agents, lipid-lowering drugs, insulin sensitizers, and other western medicines.^[4]^ However, these types of treatments have limitations, insufficient effectiveness, and potential side effects such as liver and kidney function damage, recurrence after drug withdrawl^.[3,5]^ Due to the complexity of NAFLD’s causes, its treatment is multifaceted. In China, various applications of traditional Chinese medicine (TCM) are among mainstream treatment methods for NAFLD. Given the similarity of CHD and NAFLD, they can be treated altogether. Shenxiang Suhe Pills has not yet been studied for the therapy of NAFLD, but there is some research supporting its effectiveness in the management of CHD. This study intends to intervene in the development of NAFLD at different targets and assess its clinical effectiveness, taking into account not only the pathology of NAFLD but also complications such as CVD. We used Shenxiang Suhe Pills in combination with western treatment to treat patients with CHD and NAFLD, and evaluated its clinical efficacy.

## 2. Patients and methods

### 2.1. Patients

A total of 56 patients with CHD and NAFLD who were admitted in Hangzhou Normal University Affiliated Hospital from January 2021 to December 2021 were selected and randomly divided into an experimental group and a control group, with 28 in each group. During the observation and follow-up period, a total of 5 cases dropped out (2 cases in the experimental group and 3 cases in the control group). The other 51 subjects remained in the study, and there was no statistical difference in the general data between the 2 groups (*P* > .05) (Table 1). This study has been reviewed and approved by the Ethics Committee of Hangzhou Normal University Affiliated Hospital (2019-HS-44).

**Table 1:** Comparison of general information data between the 2 groups.

Items	Experimental group (n = 26)	Control group (n = 25)	*P* value
Age (years)	56.69 ± 8.49	56.92 ± 10.77	.933
Gender (male/female)	16/10	12/13	.331
Height (cm)	163.73 ± 7.18	161.32 ± 10.42	.339
Weight (kg)	67.96 ± 10.48	67.76 ± 7.83	.938
BMI (kg/m^2^)	25.33 ± 3.34	26.40 ± 5.74	.419
History of hypertension (case)	16	21	.072
History of diabetes (case)	11	11	.903
Smoking history (example)	9	10	.691

### 2.2. Diagnostic criteria

#### 2.2.1. *CHD.* ^[7,8]^

Inclusion criteria: based on clinical symptoms, dynamic evolution of electrocardiogram, and relevant tests and examinations of myocardial markers, at least 1 coronary artery stenosis was confirmed by coronary angiography (CAG) to be greater than or equal to 50%, and percutaneous coronary intervention was performed or coronary artery bypass grafting therapy.

#### 2.2.2. *Exclusion criteria.*

① Obvious heart valve disease.
② Severe liver failure and renal failure.
③ Traumatic chest pain.
④ Malignant tumor.
⑤ Hematological diseases.
⑥ Acute inflammatory diseases and chronic inflammatory diseases (COPD).
⑦ history of major surgical trauma within 6 months.
⑧ autoimmune disease.

#### 2.2.3. *CAG*.

All selected patients underwent routine CAG, left coronary artery and CAG by Judkins puncture method, 6 projection positions to examine the left coronary artery, and 2 projection positions to examine the right coronary artery. The results of CAG were analyzed by 2 experienced interventional cardiologists.

#### 2.2.4. *NAFLD*.

NAFLD diagnostic criteria in the “Guidelines for the Diagnosis and Treatment of Non-Alcoholic Fatty Liver Diseases “(2018 revised edition) of the by the Fatty Liver and Alcoholic Liver Disease Group of the Chinese Medical Association Hepatology Branch^[3]^

1. No history of drinking or excessive drinking: in the past 12 months, the ethanol content was < 210 g/week for males and < 140 g/week for females.
2. No use of amiodarone, methotrexate, or tamoxifen.
3. Excluding certain conditions that may lead fatty liver, such as genotype 3 HCV infection, total parenteral nutrition, hepatolenticular degeneration, autoimmune liver disease, *β*-lipoprotein deficiency, celiac disease, and some insulin resistance-related syndromes.
4. Liver biopsy is required according to diagnosis, treatment, and prognosis, and liver histological changes meet the pathological criteria for the diagnosis of fatty liver disease. In this study, the imaging evidence of fatty liver was diagnosed by *B*-ultrasound.

### 2.3. *Exclusion criteria*

① Patients who do not meet the TCM diagnosis of Qi and Yin deficiency or blood stasis obstruction syndrome.
② Heavy drinkers: drinking for more than 5 years, the ethanol content of men and women is ≥ 40g/day, ≥ 20g/day, respectively, or ethanol daily intake ≥ 80g/day during 2 weeks.
③ Women who are allergic to any ingredients in the drug in the past, and pregnant and lactating women.
④ Complicated with short-term progressive diseases, such as cancer, active hepatitis, severe infection, blood system diseases, etc.

### 2.4. Randomizing method

A case–control study was conducted in Hangzhou Normal University Affiliated Hospital. According to the inclusion and exclusion criteria, patients with CHD and NAFLD were enrolled in the hospital. After obtaining the informed consent from the patients, the random number table was searched according to the order by the patients were enrolled, so that the patients were divided into the experimental group and the control group.

### 2.5. Treatment methods

#### 2.5.1. Lifestyle guidance.

Diet: low salt and fat, daily calorie intake shall be less by 500 kcal/day than original total daily intake. Exercise: 3 times or more exercise every week, with total time for 150 minutes or more.

#### 2.5.2. Drug treatment

Both groups were treated by routine antiplatelet, angiotensinase-converting enzyme inhibitor or angiotensin II receptor antagonist, beta-blocker, statin (statin drugs with doses needed to moderately reduce cholesterol). The experimental group was given Shenxiang Suhe Pill (0.7g po bid), in addition to the medicines taken by the control group. Four weeks as a course of treatment, and 3 courses of treatment were given.

#### 2.5.3. Shenxiang Sohe Pill

Manufacturer: Hangzhou Huqing Yutang Pharmaceutical Co., Ltd.

Specification: 0.7 g/pill

Dosage form: pill

Approval number: National Medicine Zhunzi Z33020141.

### 2.6. Observation indicators

#### 2.6.1. NAFLD degree evaluation

Before and after 12 weeks from the treatment, FibroScan was used to measure controlled attenuation parameter (CAP), and the severity of fatty liver was divided into 4 grades according to the value (Consensus Opinions on Diagnosis and Treatment of Nonalcoholic Fatty Liver Disease with Integrated Traditional Chinese and Western Medicine“ [2017])^[9]^

1. Normal: CAP < 238 db/m;
2. Mild fatty liver: 238 db/m ≤ CAP < 259 db/m;
3. Moderate fatty liver: 259 db/m ≤ CAP < 292 db/m;
4. Severe fatty liver: CAP ≥ 292 db/m.

#### 2.6.2. NAFLD treatment effect evaluation

1. Recov ery: CAP < 238db/m; Liver enzyme alanine aminotransferase (ALT) index returned to normal;
2. Markedly effective: CAP grade decreased from severe to mild; ALT decreased by > 50%;
3. Effective: CAP grading is reduced from severe to moderate/moderate to mild; ALT decline is in the range of 30% to 50%;
4. Invalid: CAP grade increased or did not change; ALT decreased degree < 30%.
5. Total effective rate = (recovery + markedly effective + effective)/n

### 2.7. Laboratory indicators

Laboratory tests: collecting blood samples from cubital vein (fasting for more than 10 hours in the morning before collection), which than were used to detect blood inflammatory indicators (lactate dehydrogenase [LDH], high sensitivity-reactive protein [hs-CRP]), liver function (ALT, aspartate aminotransferase [AST]) and blood lipids (triglyceride [TG], total cholesterol [TC], LDL-c, HDL-c) and other indicators.

### 2.8. Statistical methods

Statistical analysis was performed using SPSS 26.0 software. After arranging the collected data, the measurement data were tested for normality. For data that conformed to the normal distribution, the paired *t* test was used for comparison before and after the treatment, and the *t* test was used for inter-group comparison; the non-parametric test was used for the non-normal data. The rank sum test was used for the orderly leveled data. The inter-group difference of count data was compared by the *Χ*^2^ test. The testing level is set as *α* = 0.05, with *P *< .05 as the difference being statistically significant.

## 3. Results

### 3.1. Comparison of clinical efficacy between the 2 groups of patients

**Figure 1.**
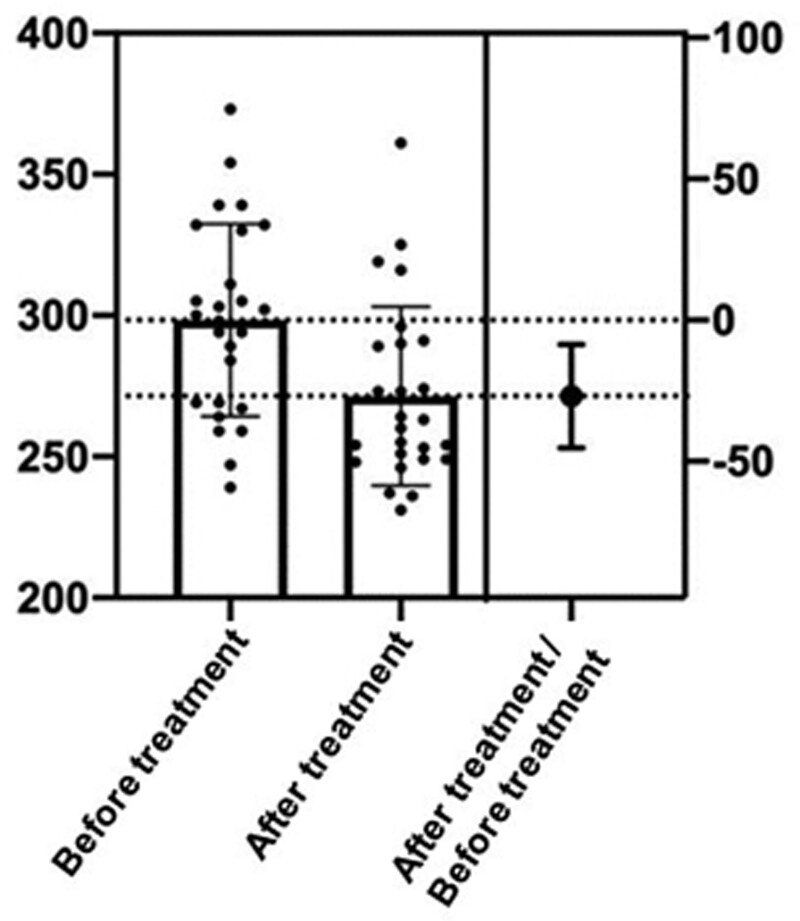
Differences of CAP values in the experimental group before and after treatment. CAP = controlled attenuation parameter.

**Figure 2.**
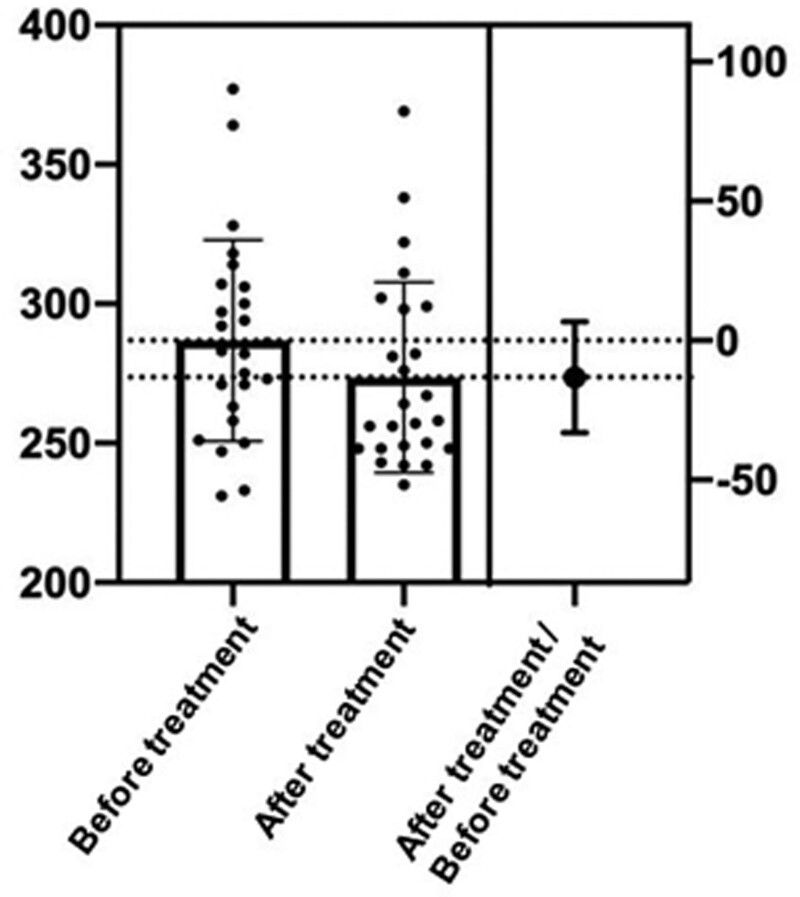
Differences of CAP values in the control group before and after treatment. CAP = controlled attenuation parameter.

Compared with the control group, the CAP value of the experimental group decreased more significantly, and the severity classification of NAFLD was also significantly improved (*P* < .05). And compared with the control group, the treatment effect of the experimental group was more effective (*P* < .05). The results are shown in Tables 2–4 and Figures 1 and 2.

### 3.2. Comparison of cell damage and inflammatory indicators between the 2 groups

Compared with the control group, LDH and hs-CRP in the experimental group decreased after treatment (*P* < .05). The results are shown in Tables 5 and 6.

**Table 5:** Comparison of LDH between the 2 groups.

Item	Experimental group (n = 26)		Control group (n = 25)		*P′*
	Before treatment	After treatment	Before treatment	After treatment	
LDH	198.42 ± 63.39	176.42 + 31.00*	175.88 ± 40.31	172.84 ± 39.78 ^#^	.026

Note: * *P *< .05, #*P *> .05 are the comparisons with the same group before treatment, *P′* is the comparison between the two groups after treatment.

LDH = lactate dehydrogenase.

**Table 6:** Comparison of hs-CRP between two groups [n (%)].

Item	Experimental group (n = 26)		Control group (n = 25)		*P* value
	Before treatment	After treatment	Before treatment	After treatment	
hs-CRP	14(53.85)	20 (76.92)	14(56.00)	6(24.00)	.033

Note: *P* value* *< 0.05, there is a statistical difference between the two classification methods.

hs-CRP = high sensitivity-reactive protein.

### 3.3. Comparison of blood lipid profile and liver function between the 2 groups

Compared with the control group, TG and high density lipoprotein cholesterol indicators improved more in the experimental group than in the control group (*P* < .05). ALT and AST in neither group showed significant change (*P* > .05). The results are shown in Table 7.

**Table 7:** Comparison of blood lipid profiles and liver and kidney functions between the 2 groups.

Item	Experimental group (n = 26)		Control group (n = 25)		*P′*
	Before treatment	After treatment	Before treatment	After treatment	
TG	1.88 ± 1.08	1.48 ± 0.41 ^*^	1.69 ± 0.78	1.60 ± 0.53^#^	.046
TC	4.03 ± 1.26	4.06 ± 1.03 ^#^	4.11 ± 1.12	3.91 ± 1.11^#^	.880
LDL-C	2.51 ± 1.02	2.43 ± 0.78 ^#^	2.52 ± 0.87_	2.40 ± 0.86 ^#^	.670
HDL-C	0.95 ± 0.20	1.15 ± 0.28 ^*^	0.99 ± 0.19	1.00 ± 0.16 ^#^	.004
ALT	33.19 ± 16.91	31.35 ± 16.06^#^	28.88 ± 21.47	27.04 ± 14.52^#^	.609
AST	31.65 ± 27	28.58 ± 11.26^#^	26.36 ± 14.03	24.80 ± 7.30^#^	.491

Note: *P < .05, #*P *> .05 are the comparisons with the same group before treatment, *P′* is the comparison between the two groups after treatment.

ALT = alanine aminotransferase, AST = aspartate aminotransferase, HDL-C = high density lipoprotein cholesterol, LDL-C = low density lipoprotein cholesterol, TC = total cholesterol, TG = triglyceride.

## 4. Discussion

NAFLD is a reversible disease if patients receive treatment promptly. However, without therapeutic intervention, the rate of evolving into liver cirrhosis and liver cancer is 1% to 2% and 5% to 10%, respectively.^[10]^ NAFLD is a complex systemic metabolic disease that not only increases the risk of liver-related morbidity or mortality, but also affects multiple extrahepatic organ systems. NAFLD is an independent risk factor for CHD and CVD is the main cause of death in NAFLD.^[11]^ Moreover, for NAFLD patients complicated with cardiovascular disease, MetS, and other conditions, it is necessary to pay equal attention to medication as well as diet and exercise.^[12]^ In the treatment of NAFLD, in addition to reducing liver fat deposition and avoiding the progression of NASH, the more important thing is to improve insulin resistance (IR) and prevent MetS and its related important target organ lesions.^[3]^

In TCM, NAFLD belongs to the categories of “flank pain” and “fullness.” The lesions are located in liver and are closely related to spleen. Qi deficiency, blood stasis and phlegm are the main syndromes of CHD.^[13]^ On the 1 hand, the pathogenesis of CHD is blood stasis; on the other hand, the pathogenesis of NAFLD is spleen deficiency, stagnation of liver qi, endogenous phlegm, and poor qi movement. It can be seen that the syndrome of CHD complicated with NAFLD is qi stagnation. Qi-deficiency, Qi stagnation and other qi-machine dysfunctions, as well as phlegm stagnation, will also block the qi-machine and eventually lead to blood stasis. Therefore, in the treatment of CHD and NAFLD, attention should be paid to promoting blood circulation and removing blood stasis, soothing liver and replenishing qi, and removing phlegm and dampness.^[14]^ Shenxiang Suhe Pills use musk and storax as the king medicines. Musk and storax have the effects of promoting blood circulation and dispelling blood stasis as well as warming and dispelling turbidity. Elecampane, Cyperus officinalis, and agarwood can be used for qi and blood circulation, invigorating the spleen and nourishing qi, and regulating qi and relieving depression, they were the ministerial medicines. Atractylodes nourish spleen, and dry dampness; benzoin and frankincense promote blood circulation and remove blood stasis, they were adjuvant medicines.^[15]^The whole formula invigorates the spleen and removes dampness, promotes blood circulation, and removes blood stasis. Our results showed that Shenxiang Suhe Pills could significantly improve fatty liver grades, reduce blood inflammatory indicators, lower TG, and increase high density lipoprotein cholesterol levels. Shenxiang Suhe Pills could effectively improve the severity of CHD and NAFLD. Its mechanism may be related to restoring the balance of oxidative stress, reducing inflammation, and regulating lipid metabolism.

The pathogenesis of NAFLD has not been fully elucidated. It is currently believed to be related to those factors such as IR, dyslipidemia, oxidative stress, and inflammation.^[16]^ For NAFLD patients, persistent and excessive inflammation is a leading cause of liver fibrosis, cirrhosis, or liver cancer. Effective management of the occurrence and development of inflammation can reduce the incidence of liver cirrhosis and improve the prognosis. In the present study, we showed that after 12 weeks of conventional treatment combined with Shenxiang Suhe Pills, the blood inflammatory index hs-CRP and the cell injury index LDH decreased significantly in the patients, suggesting that Shenxiang Suhe Pills exert anti-inflammatory and antioxidant effects. The mechanism underlying the anti-inflammatory and antioxidant effects of Shenxiang Suhe Pills may be related to the following components in the Pills: Musk, the king medicine of Shenxiang Suhe Pill, has a considerable effect on resisting oxygen free radical damage and protecting damaged cells. Its mechanism may be related to improving the activity of intracellular antioxidant enzymes and scavenging lipid peroxidation induced by reactive oxygen species. It can not only improve the intracellular activity of superoxide dismutase, but also scavenge reactive oxygen species and inhibit the leakage of LDH and propanediol;^[17]^ Styrax, the other king medicine of Shenxiang Suhe Pill, can reduce the levels of inflammatory factors such as interleukin and TNF-*α* in serum, the expression of excessive NO, and oxidative stress.^[18]^ Moreover, it can decrease the level of arachidonic acid, the metabolic disorder of which is involved in inflammatory response.^[19]^ it can improve the metabolic disorder of glutamine, and has the effects of detoxification, anti-oxidation, and anti-apoptosis; Elecampane, 1 of the ministerial medicines can prevent the damage caused by inflammatory factors to the body, manifested as reducing CRP and TNF-*α* in serum and inhibiting NF-*κ*B activation and NO production.^[20,21]^ In addition, elecampane can also scavenge free radicals and antioxidant by inducing heme oxygenase- 1 production.^[22]^

The major pathological change in NAFLD is fat deposition caused by disturbance of lipid metabolism in the liver.^[23]^ The lipid content in the liver is determined by the equilibrium between fat absorption, synthesis, oxidation, and excretion. In the present study, it was shown that the liver fat deposition significantly decreased in response to 12-weeks conventional treatment combined with Shenxiang Suhe Pills, and the severity of fatty liver classification was improved; blood lipid-related indicators TG decreased and HDL- C rises with the treatment. The inhibitory role of Shenxiang Suhe Pill in liver fat and lipid accumulation may be played by the components in Atractylodes that can improve IR status while promoting lipid metabolism and inhibiting hepatic fat accumulation, thereby reducing hepatic steatosis.^[10]^ In NAFLD patients, the levels of CD36 in the liver tissue increased, and the levels of apo B100 that was closely correlated with the fat content in liver tissue decreased. Atractylodes macrocephalaon polysaccharide in Atractylodes can not only increase the levels of apo B100, but also significantly reduce the levels of CD36 in liver tissue, thus improving the process of liver fat transport. It has been shown that Atractylodes macrocephalaon polysaccharide can improve pathological changes in liver tissue in rats with fatty liver by reducing the levels of TG and free fatty acids.^[24]^ Styrax exerts inhibitory effect on free fatty acid and regulates fatty acid oxidation. In addition, it can increase the content of linoleic acid, which can function to lower lipids levels and softening blood vessels. The active components of elecampane can regulate IR, reduce TG and TC, and improve liver tissue structure.^[25,26]^ Cyanophora also has the effect of regulating blood lipids and can decrease the levels of AST, ALT, TC and TG.

Prior to the delivery of the medicine in this trial, liver functions like ALT and AST were not abnormal in either group and were thus employed as safety check indicators. None of the patients had allergies, and the findings did not reveal a statistically significant difference between before and after the therapy. The overall safety of Shen xiang Suhe Pills was considered satisfactory.

In conclusion, Shenxiang Suhe Pill can reduce liver lipid deposition, reduce the severity of NAFLD, and has anti-inflammatory and blood lipid-regulating effects. It has a certain clinical effect in the treatment of CHD complicated with NAFLD, providing some new ideas for scaling up the indications and new applicable population of Shenxiang Suhe Pill. However, due to the short observation period and the limitation of objective conditions, this is single-center study with a small sample size. The sample size would be expanded and the multi-center study will be conducted in future studies, and further studies will also be needed to fully elucidate the therapeutic mechanisms of Shenxiang Suhe Pill.

**Table 2:** Comparison of CAP values between the 2 groups.

Item	Experimental group		Control group		*P′*
	Before treatment	After treatment	Before treatment	After treatment	
CAP (db/m)	298.35 ± 34.06	271.42 ± 31.60 *	286.84 ± 36.02	273.64 ± 34.15*	<.001

*Note*: * *P *< .05, #*P *> .05 are the comparisons with the same group before treatment, *P′* is the comparison between the two groups after treatment.

CAP = controlled attenuation parameter.

**Table 3:** Comparison of fatty liver grades between the two groups [n (%)].

CAP rating	Experimental group (n = 26)		Control group (n = 25)		*P* value
	Before treatment	After treatment	Before treatment	After treatment	
Normal	0 (0.00)	3 (11.54)	2 (8.00)	1 (4.00)	.005
Mild	2 (7.69)	9 (34.62)	4 (16.00)	12 (48.00)	
Moderate	8 (30.77)	9 (34.62)	8 (32.00)	5 (20.00)	
Severe	16 (61.54)	5 (19.23)	11 (44.00)	7 (28.00)	

CAP = controlled attenuation parameter.

**Table 4:** Comparison of the therapeutic effectiveness between the experimental group and the control group.

Group	n	Get well	Effective	Efficient	Invalid	Total efficiency (%)	*P* value
Experimental group	26	3	5	11	7	73.10	.035
Control group	25	1	3	7	14	44.00	

## Acknowledgments

The work was supported by the Key medical disciplines of Hangzhou.

## Author contributions

**Conceptualization:** Jie Ni, Mingwei Wang.

**Data curation:** Jie Ni, Shenghui Zhang, Jingjie Jiang, Chunyi Wang.

**Formal analysis:** Jie Ni, Chen Chen, Jiake Tang, Wen Wen, Xingwei Zhang.

**Funding acquisition:** Mingwei Wang.

**Investigation:** Mingwei Wang.

**Methodology:** Jie Ni.

**Supervision:** Mingwei Wang.

**Writing – original draft:** Jie Ni, Yao You.

**Writing – review & editing:** Jie Ni, Siqi Hu, Mingwei Wang.
